# Cascade-activatable nano-prodrug amplifies ROS-RNS signaling to target NQO1-associated redox vulnerability for melanoma therapy

**DOI:** 10.1186/s12951-026-04825-0

**Published:** 2026-08-03

**Authors:** Honglin Tang, Chongzhi Wu, Wei Wang, Xi Cheng, Yong Dong, Zhiyuan Gao, Pengfei Chen, Xin Xie, Yong Huang, Lihe Sun, Bowen Li, Da Li

**Affiliations:** 1 Department of Medical Oncology Sir Run Run Shaw Hospital School of Medicine, Zhejiang University, Hangzhou, 310058 China; 2 School of Pharmacy, Anhui Medical University, Hefei, 230032 P. R. China; 3 School of Pharmacy, Guangdong Medical University, Dongguan, 523808 China

**Keywords:** Ferroptosis, ROS–RNS Cascade, Nano-Prodrug, GSH-Responsive Nanoplatform, Peroxynitrite (ONOO⁻)

## Abstract

Ferroptosis, an iron-dependent form of regulated cell death driven by lipid peroxidation (LPO), has emerged as a promising strategy for cancer therapy but remains limited by insufficient hydrogen peroxide (H₂O₂), restricted catalytic iron availability, and the extremely short lifetime of hydroxyl radicals (·OH) in the tumor microenvironment. Here, we report a self-assembled nano-prodrug designed to amplify oxidative stress through coordinated reactive oxygen species (ROS)-reactive nitrogen species (RNS) cascades. Transcriptomic analyses of melanoma cohorts revealed that the redox enzyme NAD(P)H: quinone oxidoreductase-1 (NQO1) is frequently upregulated and associated with oxidative stress and ferroptosis-related transcriptional programs, suggesting a potential redox vulnerability that can be therapeutically exploited. Guided by this observation, a glutathione-responsive nanoplatform (HFSAL) was constructed from a disulfide-bridged ferrocene–arginine scaffold (Fc-SS-Arg) with β-lapachone (β-lapa) co-loaded and hyaluronic acid coating for tumor-targeted delivery. Upon intracellular activation, β-lapa undergoes NQO1-mediated redox cycling to generate H₂O₂, which is further converted into ·OH through ferrocene-catalyzed Fenton reactions. Meanwhile, arginine-derived nitric oxide (NO) reacts with ROS intermediates to form highly reactive peroxynitrite (ONOO⁻), thereby amplifying oxidative damage and extending the effective range of redox stress. In addition, NO-mediated vascular remodeling alleviates tumor hypoxia and enhances intratumoral drug accumulation. Consequently, the HFSAL nano-prodrug effectively induces synergistic ferroptosis and apoptosis, leading to significant tumor growth inhibition and metastasis suppression in vivo with favorable biosafety. This work presents a redox-amplifying nano-prodrug strategy that exploits NQO1-associated metabolic vulnerability to enhance ferroptosis-based cancer therapy.

**Graphical Abstract:**

**Figure:**
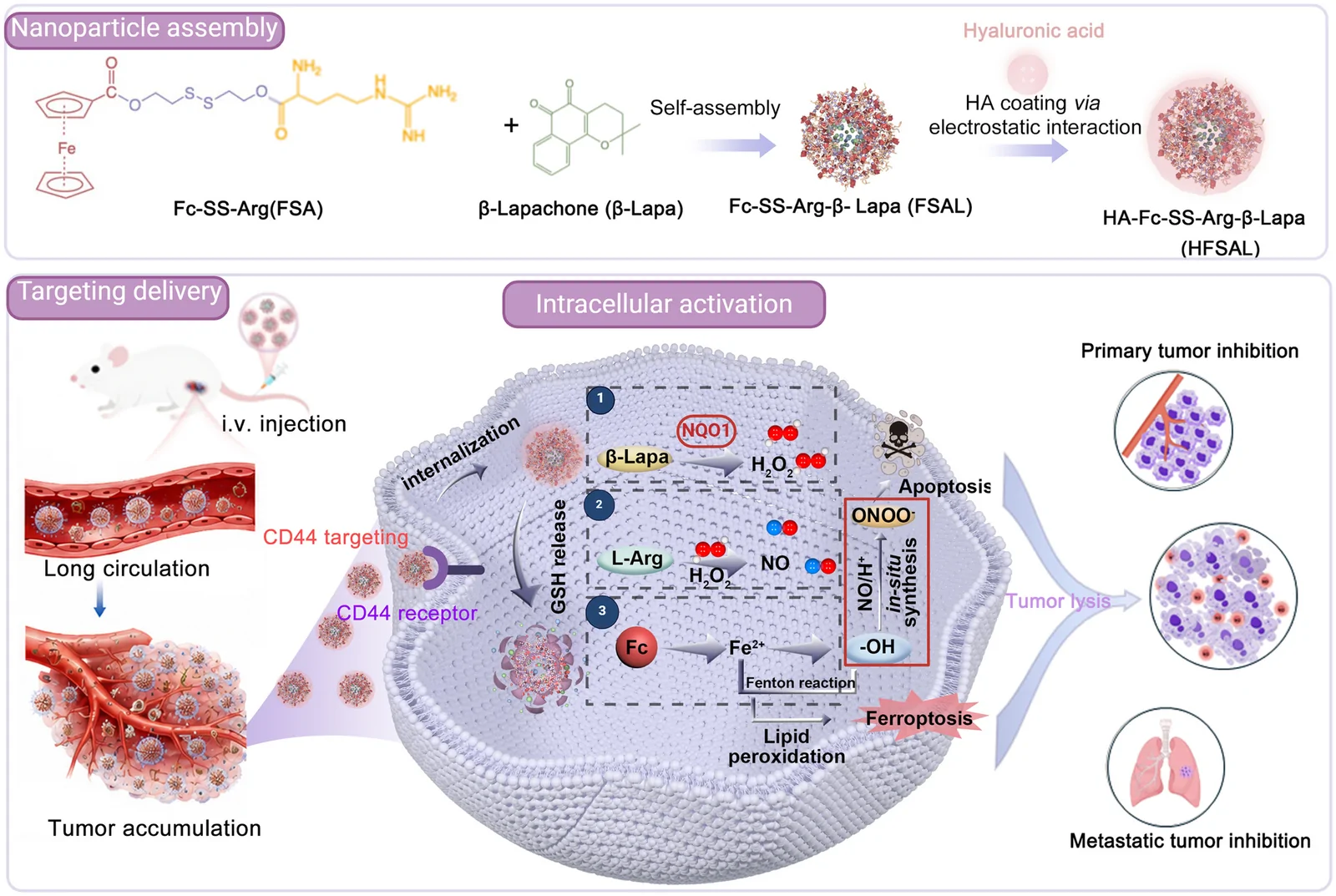

**Supplementary Information:**

The online version contains supplementary material available at https://doi.org/10.1186/s12951-026-04825-0.

## Introduction

Ferroptosis is a distinct, iron-dependent form of regulated cell death characterized by the accumulation of lipid peroxides (LPOs) and reactive oxygen species (ROS), ultimately leading to catastrophic membrane damage [1, 2]. A defining feature of ferroptosis is its reliance on Fenton chemistry, in which ferrous ions (Fe²⁺) catalyze the conversion of hydrogen peroxide (H₂O₂) into highly reactive hydroxyl radicals (·OH), thereby initiating oxidative stress–mediated cell death [3, 4]. Compared with conventional apoptosis-based therapies, ferroptosis exhibits unique advantages, including reduced susceptibility to drug resistance and improved tumor selectivity, making it an attractive strategy for cancer treatment [5–7]. Accumulating evidence indicates that ferroptosis is tightly regulated by multiple oncogenic and metabolic signaling pathways in cancer. Among them, the PI3K/AKT/mTOR pathway has been shown to modulate cellular redox homeostasis and ferroptosis susceptibility through regulation of lipid metabolism and antioxidant responses. In addition, the NRF2-GPX4 axis and iron metabolism-associated networks play central roles in maintaining ferroptotic resistance by controlling lipid peroxide detoxification and intracellular iron availability. Recent studies, including Chrysotoxine-mediated ferroptosis induction, further highlight the therapeutic potential of targeting these regulatory pathways to sensitize tumor cells to ferroptotic cell death [8]. However, the therapeutic efficacy of ferroptosis remains constrained by several intrinsic limitations. The cytotoxicity of ·OH is restricted by its extremely short half-life, limited diffusion distance, and rapid scavenging by intracellular antioxidants such as glutathione (GSH) [9–10]. Consequently, ROS-mediated damage is often spatially confined and insufficient to eradicate tumors. Emerging evidence suggests that integrating reactive nitrogen species (RNS) into redox-based therapy may overcome this limitation. In particular, the reaction between ROS intermediates and nitric oxide (NO) can generate peroxynitrite (ONOO⁻), a highly reactive species with stronger oxidative capacity, longer lifetime, and enhanced diffusion properties, thereby amplifying oxidative stress and extending cytotoxic effects [11, 12]. These observations highlight ROS–RNS coupling as a promising strategy to potentiate ferroptosis.

Another major challenge lies in the insufficient availability of key substrates within the tumor microenvironment (TME). Endogenous H₂O₂ levels in tumors are typically too low to sustain efficient Fenton reactions, while iron is often sequestered in storage forms, limiting the labile Fe²⁺ pool required for lipid peroxidation [13–18]. To address these constraints, β-lapachone (β-lapa), a quinone compound bioactivated by NAD(P)H: quinone oxidoreductase 1 (NQO1), has been widely explored as a tumor-selective ROS amplifier [19, 20]. Unlike conventional receptor-mediated targeting strategies, such as folate receptor-directed drug delivery, which primarily depend on differential receptor expression between tumor and normal tissues, β-lapa exploits a tumor-associated metabolic vulnerability through NQO1-dependent bioactivation [21–22]. Given the heterogeneous expression and variable accessibility of cell-surface receptors in solid tumors, metabolic activation strategies may provide an additional level of selectivity after nanoparticle internalization. Therefore, NQO1-responsive therapeutics represent a complementary approach to conventional targeting systems by coupling tumor-selective activation with enhanced intracellular ROS generation. Through futile redox cycling, β-lapa generates substantial amounts of H₂O₂ in NQO1-overexpressing tumor cells, thereby enhancing oxidative stress. Meanwhile, ferrocene (Fc), an organometallic Fe²⁺ donor, provides a stable and tunable source of catalytic iron for Fenton reactions [23–26]. Despite these advantages, the poor aqueous solubility and limited bioavailability of Fc restrict its in vivo applications, necessitating the development of effective delivery strategies [27–29].

**Fig. 1:**
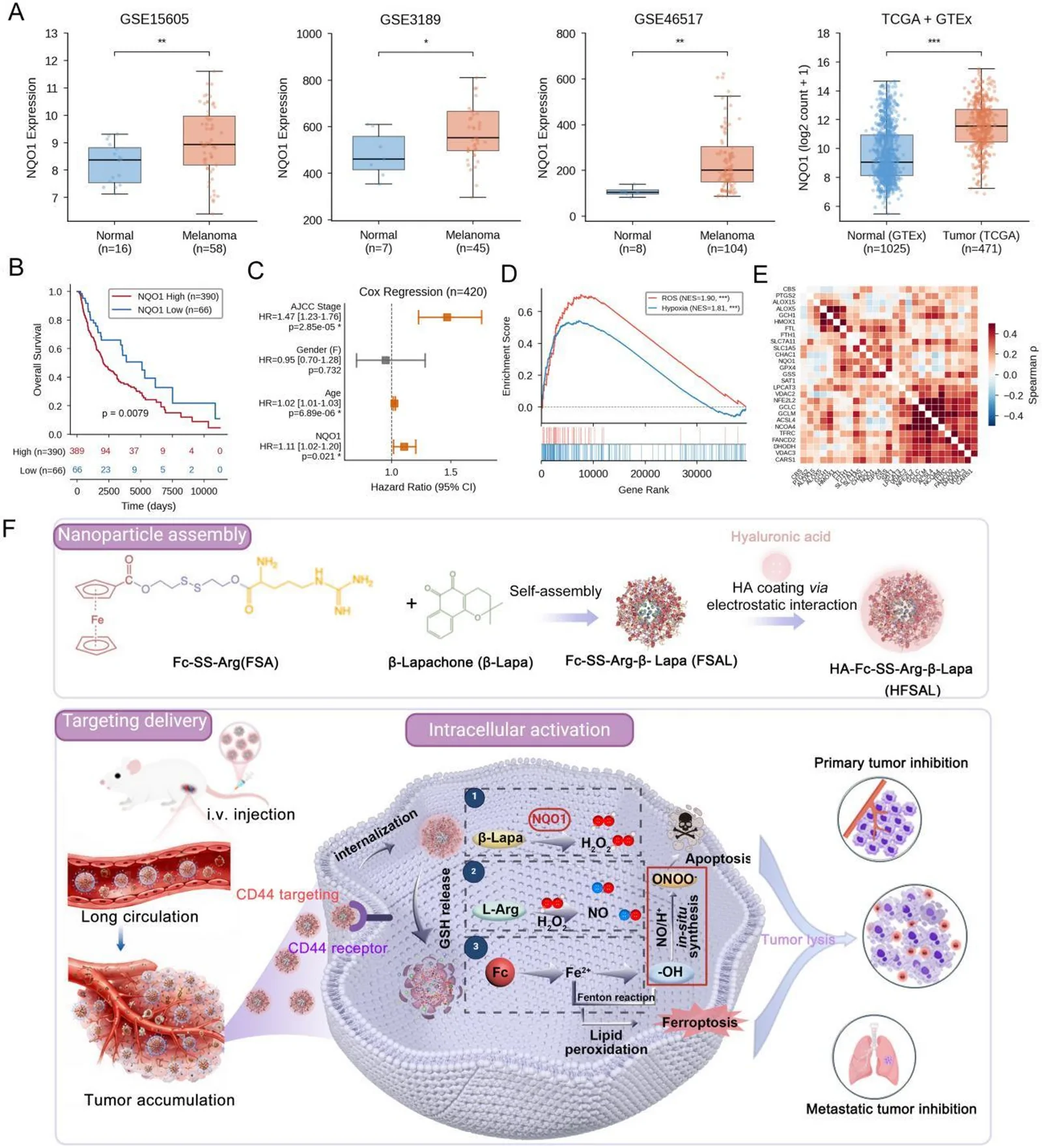
NQO1-associated redox vulnerability in melanoma and design of a ROS-RNS cascade nano-prodrug. (**A**) Expression levels of NQO1 in melanoma and normal tissues across multiple transcriptomic datasets, including GSE15605, GSE3189, GSE46517, and TCGA-SKCM integrated with GTEx normal samples. Data are presented as box plots with indicated sample sizes; statistical significance was determined by unpaired two-tailed Student’s t-test. (**B**) Kaplan–Meier overall survival analysis of melanoma patients in the TCGA-SKCM cohort stratified by high and low NQO1 expression. (**C**) Multivariate Cox regression analysis of overall survival in the TCGA-SKCM cohort, showing the hazard ratios (HRs) and 95% confidence intervals for NQO1 expression and clinical variables. (**D**) Gene set enrichment analysis (GSEA) comparing NQO1-high and NQO1-low tumors, showing enrichment of oxidative stress–related pathways, including reactive oxygen species (ROS), hypoxia, and angiogenesis. (**E**) Correlation heatmap of NQO1 expression with ferroptosis-related genes in the TCGA-SKCM dataset. Color intensity represents the strength of correlation. (**F**) Schematic illustration of the design, tumor-targeted delivery, and intracellular activation mechanism of HFSAL. HFSAL was prepared through the self-assembly of Fc-SS-Arg (FSA) and β-lapachone (β-Lapa), followed by hyaluronic acid (HA) coating. After intravenous administration, HA coating prolongs blood circulation and facilitates tumor accumulation through the enhanced permeability and CD44-mediated targeting. Following cellular internalization, elevated intracellular glutathione (GSH) triggers nanoparticle disassembly and cargo release. β-Lapa undergoes NQO1-mediated redox cycling to generate H_2_O_2_, while ferrocene (Fc) provides Fe^2+^ to catalyze Fenton reactions and produce hydroxyl radicals (·OH). Simultaneously, L-arginine (L-Arg) promotes nitric oxide (NO) generation. The interaction between ROS and NO leads to peroxynitrite (ONOO^−^) formation, resulting in ROS-RNS cascade amplification. These processes synergistically induce ferroptosis and apoptosis, promote vascular normalization, and ultimately inhibit primary tumor growth and metastatic progression

Based on these considerations, the integration of ROS amplification, Fe²⁺ supplementation, and RNS generation within a unified platform represents a rational strategy to overcome the rate-limiting barriers of ferroptosis therapy. Herein, we report a GSH-responsive, self-assembled nano-prodrug (HFSAL) that enables the co-delivery of Fc, β-lapa, and L-arginine (L-Arg) to orchestrate a cascade ROS-RNS amplification process (Fig. 1). This nanoplatform is constructed via the self-assembly of a disulfide-linked Fc–Arg conjugate, followed by β-lapa loading and hyaluronic acid (HA) coating to enhance tumor targeting *via* CD44-mediated uptake. Compared with conventional stimulus-responsive delivery systems, such as spermine-decorated pH-responsive nanostructures, HFSAL integrates HA-mediated tumor targeting, GSH-triggered nanoparticle disassembly, and NQO1-directed intracellular bioactivation, thereby providing multiple levels of tumor selectivity and therapeutic specificity beyond drug release control alone. Upon tumor accumulation and intracellular activation, β-lapa amplifies H₂O₂ production through NQO1-dependent redox cycling, while Fc catalyzes Fenton reactions to generate ·OH. Concurrently, L-Arg serves as a substrate for NO production, enabling the formation of ONOO⁻ through ROS-RNS coupling. In addition to inducing ferroptosis and apoptosis, NO-mediated signaling contributes to vascular normalization, thereby improving tumor perfusion and facilitating nanoparticle penetration. Collectively, this cascade-activatable nano-prodrug addresses three major bottlenecks of ferroptosis-based therapy-insufficient ROS generation, limited Fe²⁺ availability, and poor tumor selectivity-providing a synergistic and translatable strategy for the treatment of melanoma and other aggressive cancers.

## Materials and methods

### Materials and reagents

Chemical reagents and anhydrous solvents were obtained from commercial sources in standard commercial grade. Ferrocenecarboxylic acid, oxalyl chloride, 2,2-dithiodiethanol, N,N-diisopropylethylamine (DIPEA), 1-(3-Dimethylaminopropyl)-3-ethylcarbodiimide hydrochloride (EDCI·HCl), 4-Dimethylaminopyridine (DMAP), (S)-2-((tert-Butoxycarbonyl)amino)-5-((2,2,10,10-tetramethyl-4,8-dioxo-3,9-dioxa-5,7-diazaundecan-6-ylidene)amino)pentanoic acid (L-Arg(Boc)_3_), trifluoracetate (TFA) were purchased from Shanghai Aladdin Biochemical Technology Co., LTD. The solvents including dichloromethane (DCM) and dimethylsulfoxide (DMSO) were purchased from Aladdin and dried with molecular sieves. Other solvents used in this study were analytical grade reagents without further purification. The water used in the experiments was the triple-distilled water. All temperatures described in the experiments are internal reaction temperatures unless otherwise stated. NO detection kit (S0021S) was purchased from Beyotime (China). Peroxynitrite anion (ONOO^−^) detection kit (AAT-B16315) was purchased from AAT Bioquest (USA). Antibodies against hypoxia inducible factor (HIF)-1α (ab114977), CD31(ab182981) and Ki67 (ab15580) were obtained from Abcam (USA). Antibody against α-smooth muscle actin (α-SMA) (#56856) was obtained from Cell Signalling Technology (USA). D-Luciferin, Sodium Salt (#40901ES02) and Terminal deoxynucleotidyl transferase dUTP nick end labeling (TUNEL) Apoptosis Assay Kit (#40307ES50) were purchased from Yeasen (China). Reactive Oxygen Species Assay Kit (S0033M), 3- (4,5)-dimethylthiahiazo (-z-y1)-3,5-di- phenytetrazoliumromide (MTT) Cell Proliferation and Cytotoxicity Assay Kit (C0009S) were purchased from Beyotime (China).

### Mice and cell culture

C57BL/6 mice (female, 6–8 weeks old) were purchased from Beijing Biocytogen and maintained in the Animal Center of Zhejiang University under specific pathogen-free (SPF) conditions, with a 12-h light/12-h dark cycle and free access to food and water. All animal experiments were conducted in accordance with the guidelines of the Animal Research Committee of Zhejiang University. The murine melanoma cell line B16F10 and the murine hepatocellular carcinoma cell line Hepa1-6 were obtained from the American Type Culture Collection (ATCC, VA, USA). Cells were cultured in Dulbecco’s Modified Eagle’s Medium (DMEM) supplemented with 10% fetal bovine serum (FBS) in a humidified incubator with 5% CO₂ and 95% air at 37 °C. Routine mycoplasma testing was performed every three months using the MycoAlert Mycoplasma Detection Kit (Lonza, MD, USA).

### Bioinformatics analysis

RNA sequencing data (STAR-Counts, GDC harmonized) and corresponding clinical annotations for the TCGA-SKCM cohort were obtained from the UCSC Xena platform. To minimize batch effects, TCGA tumor samples were integrated with GTEx normal skin tissues using the TOIL reprocessed RNA-seq compendium. After filtering by sample type barcodes (− 01 and − 06), a total of 471 melanoma samples were included, and 1,025 sun-exposed normal skin samples from GTEx were used as controls. Three independent GEO datasets (GSE15605, GSE3189, and GSE46517) were downloaded as SOFT files, and NQO1 expression was extracted using probe sets (201467_s_at, 201468_s_at, 210519_s_at) and averaged per sample.

NQO1 expression differences between melanoma and normal tissues were evaluated using one-tailed Mann–Whitney U tests based on the a priori hypothesis of upregulation in melanoma. The optimal cutoff for survival stratification was determined using the surv_cutpoint function (R package survminer), and Kaplan–Meier analysis with log-rank test was performed. Multivariate Cox regression analysis was conducted to assess the independent prognostic value of NQO1 expression, adjusting for age, gender, and AJCC tumor stage, with only complete cases included.

For pathway analysis, genes were ranked according to log2 fold change between NQO1-high and NQO1-low groups and subjected to preranked gene set enrichment analysis (GSEA) using the GSEApy package with the MSigDB Hallmark gene sets. Normalized enrichment scores (NES) and FDR-adjusted q-values were calculated to determine significant pathways.

To explore the association between NQO1 and ferroptosis, Spearman correlation analysis was performed between NQO1 and 26 core ferroptosis-related genes curated from the FerrDb database across TCGA-SKCM samples. Correlation coefficients (Spearman’s ρ) were visualized using hierarchical clustering–based heatmaps.

### Synthesis of Fc-SS-OH (FSO)

Ferrocenecarboxylic acid (460 mg, 2 mmol) was dissolved in anhydrous DCM (10 mL) in a flask, and then oxalyl chloride (850 µL, 10 mmol) was added dropwise to the mixture under N_2_ atmosphere. The reaction solution was stirred at room temperature for 3 h to ensure full activation. Subsequently, the mixture was rotary evaporated to remove residual oxalyl chloride and redissolved in anhydrous DCM (5 mL), and then added dropwise to the solution of 2,2-dithiodiethanol (924 mg, 6 mmol) and DIPEA (900 µL, 4 mmol) in anhydrous DCM (5 mL). The reaction was stirred at room temperature for another 3 h. The resulted mixture was extracted with DCM (150 mL × 3). The combined organic layer was washed with brine (150 mL × 3), dried over Na_2_SO_4_ and filtered. The filtrate was concentrated under reduced pressure and purified by column chromatography with MeOH/DCM (1/100) as eluent to obtain FSO (589 mg, 80.5% yield) as brownish-yellow oily liquid. ^1^H NMR (400 MHz, CDCl_3_, Figure S1) δ = 4.84 (t, J = 1.9 Hz, 2 H), 4.52 (t, J = 6.5 Hz, 2 H), 4.44 (t, J = 1.9 Hz, 2 H), 4.24 (s, 5 H), 3.95 (t, J = 5.8 Hz, 2 H), 3.51 (s, 1H), 3.06 (t, J = 6.5 Hz, 2 H), 2.96 (t, J = 5.7 Hz, 2 H).

### Synthesis of Fc-SS-Arg (Boc)

Under N_2_ atmosphere, L-Arg (Boc)_3_ (237 mg, 0.5 mmol), EDCI·HCl (144 mg, 0.75 mmol), and DMAP (73 mg, 0.6 mmol) were dissolved in anhydrous DCM (5 mL). FSO (183 mg, 0.5 mmol) was added on an ice bath. After 10 min, the mixture was transferred to room temperature and stirred for 12 h. Finally, the resulting mixture was dried under reduced pressure and purified by column chromatography with EA/PE (1/10) as eluent to obtain Fc-SS-Arg (Boc) (224 mg, 54.5% yield) as brownish-yellow oily liquid. ^1^H NMR (400 MHz, CDCl_3_, Figure S2) δ = 11.49 (s, 1H), 8.36 (s, 1H), 4.82 (t, J = 1.8 Hz, 2 H), 4.50–4.47 (m, 3 H), 4.42 (s, 3 H), 4.34 (s, 1H), 4.23 (s, 6 H), 3.46 (s, 2 H), 3.06–3.02 (m, 2 H), 3.00-2.97 (m, 2 H), 2.05 (s, 1H), 1.92 (s, 1H), 1.69 (m, 3 H), 1.51 (s, 9 H), 1.46 (s, 9 H), 1.34 (s, 1H), 1.29 (s, 1H), 1.26 (s, 2 H), 0.96 − 0.92 (m, 1H), 0.87 − 0.85 (m, 2 H). HR-MS (ESI, Figure S3): calculated for C36H55FeN4O10S2+ ([M + H]^+^) (m/z) 823.27035, found 823.26694.

### Synthesis of Fc-SS-Arg (FSA)

Fc-SS-Arg (Boc) (206 mg, 0.25 mmol) was dissolved in anhydrous DCM (4 mL), to which TFA (1 mL) was added dropwise. Subsequently, the mixture was rotary evaporated to remove residual TFA. The resulted mixture was extracted with EA (150 mL × 3). The combined organic layer was washed with brine (150 mL × 3), dried over Na_2_SO_4_ and filtered. The filtrate was concentrated under reduced pressure and purified by column chromatography with EA/PE (1/10) as eluent to obtain FSA (95 mg, 72.8% yield) as brownish-yellow oily liquid. ^1^H NMR (400 MHz, CDCl_3_, Figure S4) δ = 4.84 (t, J = 1.9 Hz, 1H), 4.52 (t, J = 6.5 Hz, 1H), 4.44 (t, J = 2.0 Hz, 2 H), 4.23 (d, J = 7.5 Hz, 4 H), 3.95 (t, J = 5.8 Hz, 1H), 3.06 (t, J = 6.5 Hz, 1H), 2.96 (t, J = 5.7 Hz, 1H), 2.64 (s, 2 H), 1.35 (s, 1H), 1.31–1.28 (m, 9 H), 0.91-0.83 (m, 3 H), 0.09 (s, 3 H). HR-MS (ESI, Figure S5): calculated for C21H31FeN4O4S2+ ([M + H]^+^) (m/z) 523.11307, found 523.11212.

### Preparation and characterization of Fc-SS-Arg@β-Lapa (FSAL) and HA-coated Fc-SS-Arg@β-Lapa (HFSAL)

Firstly, the FSA nanoparticles were prepared using a simple nanoprecipitation method. Briefly, FSA (2 mg) was dissolved in DMSO (0.25 mL) and then added dropwise into double-distilled water (4.75 mL) under ultrasonic vortex bath conditions for 20 min. During this process, the amphiphilic FSA molecules spontaneously self-assembled into nanoparticles driven by hydrophobic interactions. The resulting crude FSA nanoparticles (single-component self-assembled nano-prodrug) were further purified by dialysis against double-distilled water for 24 h using a dialysis tube (MWCO = 1000 Da), with the dialysis medium replaced every 6 h. After that, FSAL nanoparticles were prepared in a similar manner. Briefly, β-lapa (2 mg) was co-dissolved in DMSO (0.25 mL), while other procedures remained the same as those conducted in preparing FSA nanoparticles. After purification by dialysis, FSAL was lyophilized and stored at -20 ℃ for future use. To further modify the surface charge of nano-prodrug, the FSAL resuspension (2 mg/mL in 1 mL double-distilled water) was slowly added to a hyaluronic acid (HA) solution (1 mg HA in 4 mL double-distilled water) under vigorous stirring at room temperature and co-incubated for 30 min to obtain HA-coated nanoparticles HFSAL based on electrostatic attraction.

The particle morphology, particle size, size distribution and zeta potential of different formulations were examined using transmission electron microscopy (TEM) and dynamic light scattering (DLS). Additionally, the drug loading capacities of β-lapa in nano-prodrug (for FSAL or HFSAL) were further determined by high-performance liquid chromatography (HPLC), and the Fc content was quantified by measuring Fe concentration with inductively coupled plasma optical emission spectrometry (ICP-OES) analysis. Furthermore, the stability of FSAL and HFSAL during 15 days of storage under physiological conditions (simulated with PBS containing 10% FBS) was confirmed by DLS.

With or without GSH stimulation, the drug release behavior of β-lapa was evaluated. Briefly, nano-prodrug HFSAL was placed into a dialysis bag (MWCO = 1000 Da) and dialyzed against double-distilled water containing different concentrations of GSH (0, 0.05, 0.1, 1, 10 mM) at 37 °C using a shaker. At predetermined time points (0, 0.5, 1, 2, 3, 4, 5, 6 h), dialysate sample was collected and immediately replaced with an equal volume of fresh medium to maintain total dialysis volume. The release concentration of β-lapa in the collected samples was tested and calculated utilizing HPLC with a C18 column operated at 25 °C. Fc release behavior was investigated through absorption measurement according to the same way and the Fc content in each sample was detected by measuring the absorbance at its characteristic absorption peak (310 nm).

To verify the in-vitro ROS-induced hydroxyl radical (·OH) generating capability, PBS, Fc, FSA and FSAL were respectively added to a methylene blue (MB) solution (10 µg/mL, pH 5.5) containing hydrogen peroxide (H_2_O_2_, 100 µM). Tert-butanol (TBA, 1 mM) was employed as the selective scavenger of ·OH. And the absorbance at 664 nm (corresponding to the characteristic absorption peak of MB) was recorded after incubation for a specific incubation duration (0, 1, 2, 5,10, 20, 30, 45 and 60 min) to monitor the degradation of MB. Additionally, the generation of ·OH from FSA at different pH environment (pH 7.4, 6.5, 5.5) was also tested following the same procedure.

To demonstrate the in-vitro NO generation capability, various formulations (PBS, Fc, L-Arg, FSAL) were incubated in PBS (×1, pH 7.4) containing H_2_O_2_ (1 mM). The NO concentrations were measured at predetermined time points (0, 0.5, 1. 2.4, 6, 8 h) using a commercial NO detection kit according to the manufacturer’s standard protocol. Briefly, different formulations and Griess reagent were dissolved in PBS. Then, the solutions were exposed to 808 nm laser (0.5 W/cm^2^) and the UV-Vis absorbance spectra of the solution were measured at different time. The amount of NO released by the micellar nanoparticles at each time point was quantified using a standard curve established with NaNO_2_ and the Griess reagent.

To confirm the in-vitro ONOO⁻ generation capability, various formulations (PBS, Fc, L-Arg, FSAL) were first incubated with GSH (1 mM) for 4 h. Then, H_2_O_2_ (1 mM) was added into the above mixtures, followed by incubation with a PBS solution (pH 8.5) containing NaHCO_3_ (15 mM) and L-tyrosine (L-tyr, 1 mM) at room temperature. ONOO⁻ generation under different conditions was assessed using fluorescence emission measurement at 420 nm.

### Intracellular lipid peroxidation detection

B16F10 tumor cells were seeded in 12-well plates at a density of 2 × 10^5^ cells per well and incubated for 24 h with PBS (control), L-Arg, β-lapa, Fc-SS-Arg (FSA), Fc-SS-Arg@β-lapa (FSAL), or HA-coated Fc-SS-Arg@β-lapa (HFSAL). After treatment, lipid peroxidation was assessed using the BODIPY 581/591 C11 dye, which is a well-established fluorescent probe for detecting lipid peroxidation in living cells. Cells were incubated with 0.5 mL of 2 µM BODIPY 581/591 C11 working solution for 30 min at 37 °C. After incubation, cells were washed twice with PBS to remove excess dye, and 2 mL of PBS was added for fluorescence observation. The reduced form of BODIPY 581/591 C11 exhibits red fluorescence with maximal excitation at 581 nm and emission at 591 nm. In contrast, upon lipid peroxidation, the dye shifts to the oxidized form, emitting green fluorescence with excitation at 488 nm and emission at 510 nm. Fluorescence microscopy was performed to capture both red and green fluorescence. The red-to-green fluorescence ratio was calculated to assess lipid peroxidation.

### Intracellular ROS detection

The ROS generation in B16F10 cells was detected according to the previous study [30]. Intracellular ROS generation was detected using a Reactive Oxygen Species Assay Kit (Beyotime Biotechnology, S0033S, China) based on the fluorescent probe DCFH-DA. Briefly, B16F10 cells were seeded in 12-well plates at a density of 2 × 10⁵ cells per well and cultured overnight at 37 °C in a humidified atmosphere containing 5% CO₂. The cells were then treated with PBS, L-Arg, β-lapa, FSA, FSAL, or HFSAL for 24 h. After treatment, the culture medium was removed, and the cells were washed twice with PBS. Subsequently, cells were incubated with DCFH-DA working solution (10 µM) at 37 °C for 30 min in the dark. After staining, cells were washed three times with PBS to remove excess probe and observed using laser scanning confocal microscopy. Fluorescence signals were detected at an excitation wavelength of 488 nm and an emission wavelength of 525 nm. For positive control experiments, cells were treated with Rosup, the ROS inducer supplied with the kit, according to the manufacturer’s instructions before DCFH-DA staining to verify the responsiveness of the fluorescence probe.

### The MTT assay

The B16F10 cells were seeded in a 96-well plate at a density of 10^4^ cells per well at 37 °C in a 5% CO_2_ incubator overnight. Then, the cell was incubated with PBS, L-Arg, β-lapa, FSA, FSAL or HFSAL at different concentrations (0, 100, 200, 300 and 400 µg/mL) and incubated for 48 h. After that, the media were replaced by 100 µL of fresh medium containing MTT and incubated for another 4 hours. Cell viability was measured according to the protocol of MTT.

To investigate the contribution of ROS, NO, ferroptosis, and apoptosis to HFSAL-induced cytotoxicity, rescue experiments were performed. B16F10 and Hepa1-6 cells were seeded in 96-well plates at a density of 1 × 10⁴ cells per well and allowed to adhere overnight. Cells were pretreated with N-acetylcysteine (NAC, 5 mM), Carboxy-PTIO (cPTIO, 100 µM), Ferrostatin-1 (Fer-1, 1 µM), or Z-VAD-FMK (20 µM) for 2 h prior to HFSAL treatment. Cells were then incubated with HFSAL (300 µg mL⁻¹) for 48 h under standard culture conditions. Cell viability was subsequently assessed using the standard MTT assay as described above.

### Intracellular NO detection

B16F10 cells were seeded in 24-well plates at a density of 1 × 10⁵ cells per well and cultured overnight at 37 °C in a humidified atmosphere containing 5% CO₂. The cells were then treated with PBS, L-Arg, β-lapa, FSA, FSAL, or HFSAL for 24 h. After treatment, the culture medium was removed and the cells were washed twice with PBS. Intracellular NO levels were detected using a Nitric Oxide Assay Kit with DAF-FM DA (Beyotime Biotechnology, S0020S, China) according to the manufacturer’s instructions. Briefly, cells were incubated with DAF-FM DA working solution (5 µM) at 37 °C for 30 min in the dark. After staining, the cells were washed three times with PBS to remove excess probe. Cell nuclei were counterstained with Hoechst 33,342 (1:1000) for 10 min. Fluorescence images were acquired using a laser scanning confocal microscope (Leica, Germany). For positive control experiments, cells were treated with the NO inducer NOup supplied with the kit according to the manufacturer’s protocol.

### Intracellular ONOO^−^ detection

B16F10 cells were seeded in 24-well plates at a density of 1 × 10⁵ cells per well and cultured overnight at 37 °C in a humidified atmosphere containing 5% CO₂. The cells were then treated with PBS, L-Arg, β-lapa, FSA, FSAL, or HFSAL for 24 h. After treatment, the culture medium was removed and the cells were washed twice with PBS. Intracellular ONOO⁻ levels were evaluated using a BBoxiProbe^®^ Peroxynitrite (ONOO⁻) Detection Kit (BestBio, China) according to the manufacturer’s instructions. Briefly, cells were incubated with the BBoxiProbe^®^ O56 working solution at 37 °C for 30 min in the dark. After staining, cells were washed three times with PBS and counterstained with Hoechst 33,342 (1:1000) for 10 min. Fluorescence images were acquired using a laser scanning confocal microscope (Leica, Germany). For positive control experiments, cells were treated with SIN-1 (3-morpholinosydnonimine hydrochloride), a well-established peroxynitrite donor, prior to probe staining.

### Blood circulation and biodistribution study

Pharmacokinetic profiles evaluation of the nano-prodrug was conducted on healthy mice after intravenously injected with ICG labeled nanoparticles (ICG-FSAL and ICG-HFSAL, equivalent ICG dose of 10 mg/kg). After that, blood (50 µL) was collected at pre-determined time points (0, 0.5, 1, 2, 4 and 6 h post-administration), and centrifugated to obtain plasma samples at 3000 rpm for 3 min. Finaly, a TECAN Spark microplate reader was utilized to quantify the ICG content in the plasma samples, with excitation and emission wavelengths of 745 nm and 780–870 nm, respectively.

In vivo imaging study of the nanoparticle distribution was performed on mice bearing subcutaneous tumors intravenously injected with free ICG or ICG labeled NPs. Anesthetized by isoflurane, the mice were photographed under IVIS spectrum imaging system at Ex/Em 788 nm/808 nm at 0, 2, 4, 6, 12, 24 and 36 h post injection. The tissues and main organs (heart, liver, spleen, lung, kidney and tumor) were collected and photographed at 36 h.

To evaluate the tumor-specific delivery of Fc-derived Fe from the nano-prodrugs, Fe accumulation in tumors was quantified using ICP-OES. B16F10 tumor-bearing mice were intravenously administered with FSAL or HFSAL (200 µL, containing equivalent Fe content of 100 µg/mouse). After that, mice were euthanized, and tumor tissues were carefully excised, weighed, and transferred into digestion vials at predetermined time points. Each tumor sample was digested with 2 mL of aqua regia (a 3:1 v/v mixture of concentrated HCl and concentrated HNO₃) at 150 °C on a hot plate for 2 h until the solution became clear. After digestion, the samples were diluted with water to a final volume of 10 mL. The Fe concentration in each digest was measured using ICP-OES with standard Fe solutions for calibration. Three mice per group were analyzed, and each sample was measured in triplicate.

### RNA sequencing analysis

For tumor RNA sequence analysis, 1 × 10^6^ B16F10 tumor cells were injected subcutaneously into the right flank of C57BL/6 mice and randomly divided into 2 groups at day 7, followed by the treatment with 200 µL PBS or HFSAL once a week for 2 weeks Total RNA was isolated from the above treated tumors. RNA purification, reverse transcription, library construction and sequencing were performed at Majorbio Bio-pharm Biotechnology Co., Ltd. (Shanghai, China) according to the manufacturer’s instructions (Illumina, San Diego, CA). The differentially expressed genes (DEG) analysis was performed using DESeq R packageGenes with a log2 fold change > 1.5 and p-value < 0.005 were considered significantly differentially expressed. Principal component analysis (PCA) was performed to visualize global transcriptomic differences between the PBS and HFSAL-treated groups. Gene Ontology (GO) pathway enrichment analyses were conducted on differentially expressed genes using the clusterProfiler R package, with false discovery rate (FDR) < 0.05 as the significance threshold.

### In vivo anti-tumor studies

C57BL/6 mice (Female, 6–8 weeks old, 16–20 g) were housed in a dark/light cycle pathogen-free environment and allowed ad libitum access to water and food. All animal experiments were conducted in accordance with the policies of animal ethics committee of Zhejiang University.

To construct B16F10 primary tumor model, 1 × 10^6^ Luci-B16F10 cells were injected subcutaneously into the right flank of C57BL/6 mice. When tumors reached the size of about 50–80 mm^3^ at day 7, the tumor-bearing mice were randomly divided into 5 groups and intravenously administration with 200 µL PBS, β-lapa, FSA, FSAL or HFSAL once a week for 2 weeks. The tumor volume, body weight and temperature of the mice were measured every three days until the end of the experiments. Tumor volume was calculated by the formula: tumor volume = 0.5 × width^2^ × length. The luciferase-based bioluminescence imaging system was also measured to assess tumor growth. About one-half of the mice were sacrificed on day 21, and the peripheral blood, major organs and tumors of mice were obtained and analyzed. The remaining mice were used for the evaluation survival rate. Mice were sacrificed when the tumor length exceeded 2 cm or when the mice became moribund. Survival was evaluated from the first day of tumor incubation until day 40.

In the lung metastases tumor model, the mice were intravenously injected with 5 × 10^5^ Luci-B16F10 cells and then randomly divided into 5 groups and administrated as described above. The lungs were harvested, photographed, fixed, and sections were taken for H&E staining on day 21. The metastasis nudes on lung surface were counted to assess the effect of anti-metastasis. Lung metastasis was further detected by ex vivo luciferase-based bioluminescence imaging. The survival rate of mice was evaluated as described above.

To assess the translational potential of HFSAL and its compatibility with immune checkpoint blockade, a B16F10 melanoma-bearing mouse model was established. When tumors reached the size of about 50–80 mm^3^, mice were randomly assigned to four groups (*n* = 5 per group): PBS, anti-PD-1, HFSAL, and HFSAL + anti-PD-1. HFSAL was administered intravenously at the same dosage and schedule used in the primary therapeutic study. Anti-PD-1 antibody (Bio X Cell, clone RMP1-14) was administered intraperitoneally at a dose of 200 µg per mouse every three days. For the combination group, mice received both HFSAL and anti-PD-1 according to the corresponding treatment schedules. Antitumor efficacy was evaluated based on tumor growth inhibition.

### Immunofluorescence

The immunofluorescence study was performed as described previously [31]. Briefly, the tumor sections were labeled with antibodies against CD31 (1:100) and α-SMA (1:100) overnight at 4 ℃ for co-localization detection. The sections were washed twice for 5 min with PBS and then incubated Alexa Fluor 488- and Alexa Fluor 594-conjugated secondary antibodies for 1 h. Finally, the sections labeled with 4ʹ,6-diamidino-2-phenylindole (DAPI) were observed by using a confocal microscopy (Carl Zeiss, Germany).

### Immunohistochemistry

For immunohistochemistry staining of HIF-1α and Ki-67, tumor tissue sections from treated groups were incubated with primary antibody against HIF-1α (1:200) or Ki67 (1:200) in a humidified chamber at 4 ℃ overnight. Then, the sections were incubated with horseradish peroxidase-conjugated secondary antibody at 37 ℃ for 1 h and reacted with 3,3’-Diaminobenzidine (DAB). Images were acquired with a phase-contrast microscope. Every fifth section was obtained for assessment.

### Western blot analysis of tumor tissues

To further validate the immunofluorescence and histological findings, western blot analysis was performed using tumor tissues collected from the B16F10 subcutaneous and lung metastatic models after treatment. Tumor tissues were homogenized in RIPA lysis buffer (Beyotime, China) supplemented with protease and phosphatase inhibitor cocktails and incubated on ice for 30 min. After centrifugation at 12,000 rpm for 15 min at 4 °C, the supernatants were collected and protein concentrations were determined using a BCA Protein Assay Kit (Thermo Fisher Scientific, USA). Equal amounts of protein were separated by SDS–PAGE and transferred onto PVDF membranes (Millipore, USA). Membranes were blocked with 5% non-fat milk in TBST for 1 h at room temperature and then incubated overnight at 4 °C with primary antibodies against HIF-1α (1:1000, CST, #36169), Ki67 (1:1000, Abcam, ab15580), cleaved caspase-3 (1:1000, CST, #9664), and GAPDH. After washing with TBST, membranes were incubated with HRP-conjugated secondary antibodies for 1 h at room temperature. Protein bands were visualized using an enhanced chemiluminescence (ECL) detection kit (Thermo Fisher Scientific, USA) and imaged using a ChemiDoc imaging system (Bio-Rad, USA). GAPDH was used as the loading control.

### Toxicity evaluation in vivo

Blood was collected from the tumor-bearing mice to evaluate the levels of lactate dehydrogenase (LDH), blood urea nitrogen (BUN), alanine aminotransferase (ALT), aspartate aminotransferase (AST), and alkaline phosphatase (ALP) in serum. To evaluate the changes in tissue morphology, the major organs from the above treated mice were dislodged and fixed in 4% paraformaldehyde. Then, stained in harris hematoxylin and eosin-phloxine B solutions, and dehydrate through alcohol.

### Statistical analysis

All data were analyzed by Graphpad prism 8.0. The overall survival rates were evaluated by Log-rank test. One-way analysis of variance (ANOVA) with a Tukey’s post-hoc test was used when more than two groups were analysed. Two-way ANOVA with a Bonferroni post-hoc test was performed when both treatment and time were compared. Unpaired two-tailed Students’t-test was unitized for the comparison of two groups. Data were displayed as mean ± standard deviation (SD). The P-value less than 0.05 was regarded as significant (*P < 0.05, **P < 0.01, ***P < 0.001, ****P < 0.0001).

## Results and discussion

### Clinical transcriptomic analyses reveal NQO1-associated redox vulnerability in melanoma

To identify potential redox-associated vulnerabilities that could be exploited for oxidative stress-based therapy, we first examined the expression pattern of NQO1 in melanoma. Analysis of multiple independent transcriptomic datasets revealed that NQO1 expression was consistently elevated in melanoma tissues compared with normal skin, including three GEO cohorts (GSE15605, GSE3189, and GSE46517) as well as the TCGA-SKCM dataset integrated with GTEx controls (Fig. 1A). We next assessed the clinical relevance of NQO1 expression in the TCGA-SKCM cohort. Kaplan–Meier survival analysis showed that patients with higher NQO1 expression exhibited a trend toward poorer overall survival compared with those with lower expression (Fig. 1B). Consistently, multivariate Cox regression analysis incorporating clinicopathological variables identified NQO1 expression as an independent prognostic factor in melanoma (Fig. 1C). These results suggest that NQO1-associated metabolic programs may contribute to melanoma progression and may represent a potential therapeutic vulnerability. To further characterize the biological processes associated with NQO1 expression, gene set enrichment analysis (GSEA) was performed by comparing NQO1-high and NQO1-low tumors within the TCGA cohort. The analysis revealed significant enrichment of multiple oxidative stress–related pathways in NQO1-high tumors, including reactive oxygen species signaling, hypoxia responses, and angiogenesis pathways (Fig. 1D), indicating that tumors with elevated NQO1 expression are associated with enhanced redox activity and altered tumor microenvironmental features. Because ferroptosis is tightly linked to oxidative stress and lipid peroxidation, we further examined the relationship between NQO1 and ferroptosis-related genes. Correlation analysis demonstrated that NQO1 expression was significantly associated with several key regulators of ferroptosis, including genes involved in iron metabolism, lipid peroxidation, and antioxidant defense (Fig. 1E), suggesting that NQO1-associated redox metabolism may influence ferroptosis susceptibility in melanoma. Collectively, these transcriptomic analyses indicate that NQO1-high melanomas represent a redox-active tumor subset characterized by enhanced oxidative stress signaling and ferroptosis-associated transcriptional programs, suggesting that strategies aimed at amplifying intracellular ROS may be particularly effective in this context. Given that β-lapachone is a well-established NQO1-bioactivatable quinone that induces hydrogen peroxide production through futile redox cycling, these findings further support NQO1-associated redox vulnerability as a therapeutic target in melanoma. This provides a mechanistic rationale for incorporating β-lapachone into redox-amplifying therapeutic strategies.

### Design, synthesis, and physicochemical characterization of the cascade-activatable nano-prodrug HFSAL

**Fig. 2:**
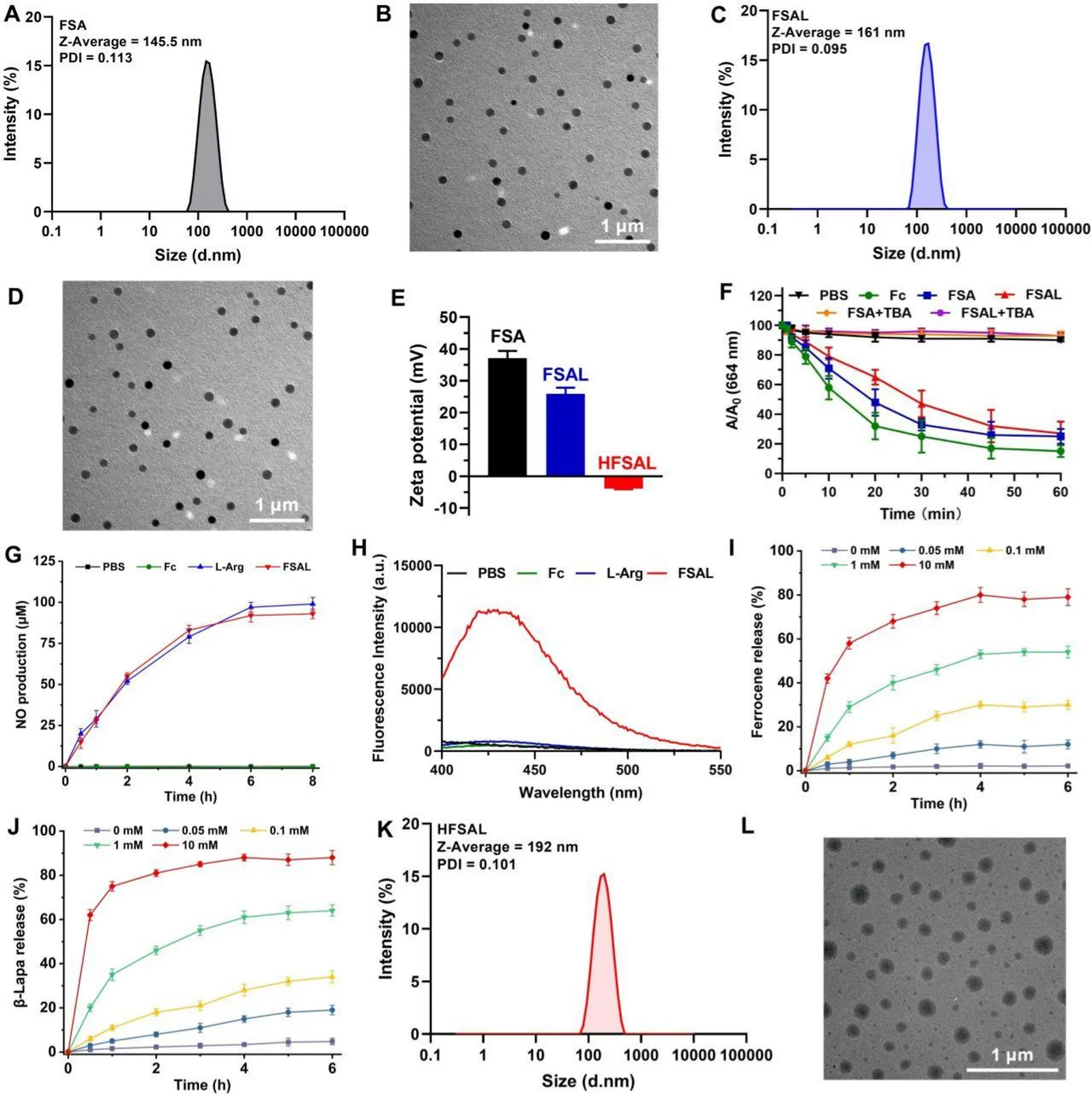
Physicochemical characterization of the nano-prodrug and evaluation of ROS-RNS generation and GSH-responsive release. (**A**) Hydrodynamic size distribution and polydispersity index (PDI) of FSA nanoparticles measured by dynamic light scattering (DLS). (**B**) Transmission electron microscopy (TEM) image of FSA nanoparticles. Scale bar: 1 μm. (**C**) Hydrodynamic size distribution and PDI of FSAL nanoparticles measured by DLS. (**D**) TEM image of FSAL nanoparticles. Scale bar: 1 μm. (**E**) Zeta potentials of FSA, FSAL, and HFSAL nanoparticles. (**F**) Hydroxyl radical (·OH) generation of different formulations evaluated by methylene blue (MB) degradation in H_2_O_2_-containing solution. (**G**) Nitric oxide (NO) production of different formulations in H_2_O_2_-containing PBS (pH 7.4). (**H**) Peroxynitrite (ONOO^−^) generation detected by fluorescence intensity changes. (**I**) GSH-responsive release behavior of ferrocene (Fc) from HFSAL nanoparticles under different glutathione concentrations. (**J**) GSH-responsive release profile of β-lapachone (β-Lapa) from HFSAL nanoparticles. (**K**) Hydrodynamic size distribution and PDI of HFSAL nanoparticles measured by DLS. (**L**) TEM image of HFSAL nanoparticles. Scale bar: 1 μm

Guided by the above clinical observations identifying NQO1-associated redox metabolism as a potential therapeutic vulnerability, we constructed a multifunctional nano-prodrug system (HFSAL) to amplify intracellular oxidative stress through coordinated ROS–RNS cascades. As illustrated in Fig. 1F, HFSAL was formed via the self-assembly of a disulfide-linked ferrocene–arginine conjugate (Fc-SS-Arg) together with β-lapa, followed by hyaluronic acid (HA) coating to improve colloidal stability and enable CD44-mediated tumor targeting. The small-molecule self-assembling prodrug FSA was synthesized via a multistep route and fully characterized by proton nuclear magnetic resonance (^1^H NMR) and high-resolution mass spectrometry (HR-MS), confirming the successful formation of the desired structure with high purity (Fig. S1-S5). Leveraging its amphiphilic nature, FSA readily formed nanoparticles through a nanoprecipitation-driven self-assembly process. As shown in Fig. 2A and B, the resulting FSA nanoparticles exhibited a uniform spherical morphology with an average hydrodynamic diameter of approximately 145.5 nm and a narrow size distribution (PDI = 0.113), as confirmed by dynamic light scattering (DLS) and transmission electron microscopy (TEM). Upon incorporation of β-lapa, the resulting FSAL nanoparticles maintained a well-defined spherical structure, with a slightly increased size (~ 161 nm) and improved uniformity (PDI = 0.095) (Fig. 2C, D). The presence of L-Arg moieties balanced hydrophilic–hydrophobic interactions within the assembly system, resulting in stable nanostructures. However, FSAL exhibited a relatively high positive ζ-potential (> 20 mV) due to surface-exposed L-Arg residues (Fig. 2E), which is unfavorable for systemic circulation. To address this limitation, HA was introduced via electrostatic interaction with L-Arg to generate HFSAL. Following HA coating, the particle size increased to ~ 192 nm, and the ζ-potential shifted to near-neutral (–4.8 mV), while maintaining excellent uniformity (PDI = 0.101) (Fig. 2E, K and L). Notably, both FSAL and HFSAL exhibited remarkable colloidal stability, with negligible changes in particle size and only minor variation in PDI after 15 days of storage (Fig. S7 and S8). These structural features collectively indicate that HFSAL is well-suited for tumor-targeted delivery and redox-responsive activation. Besides, the loading capacity of β-lapa in FSAL and HFSAL was determined by HPLC to be 78.6% and 53.4%, respectively. Moreover, the Fc content in FSAL and HFSAL was quantified as 7.58% (0.41 mmol/g) and 5.24% (0.28 mmol/g), respectively, by measuring Fe via ICP-OES.

We next evaluated the redox activity of the nanoplatform. Hydroxyl radical (·OH) generation was assessed using a methylene blue (MB) degradation assay under H₂O₂ stimulation (100 µM), mimicking tumor microenvironment conditions. Herein, MB was employed as a classical probe for ·OH. When combines with ·OH, the blue-colored MB turns colorless due to oxidative degradation, resulting in a significant decrease in absorbance at 664 nm. Negligible MB degradation was observed in the PBS group, while both Fc and Fc-containing nanoparticles (FSA and FSAL) exhibited rapid and significant MB degradation, confirming efficient ·OH production via Fenton reactions (Fig. 2F). To further validate that the observed MB degradation was specifically mediated by hydroxyl radicals (·OH), selective scavenging experiments were performed using tert-butyl alcohol (TBA), a well-established ·OH quencher. when TBA (1 mM) was co-incubated with FSA or FSAL, the degradation of MB was markedly suppressed, with the absorbance at 664 nm remaining at a level comparable to that of the PBS control group throughout the entire 60 min incubation period. These results confirm that the MB degradation induced by the Fc-containing nano-prodrugs is indeed attributable to ·OH generation. Furthermore, ·OH generation was markedly enhanced under acidic conditions (pH 6.5 and 5.5), consistent with tumor and lysosomal environments (Fig. S6), indicating preferential activation within tumor-associated conditions. In parallel, the NO-generating capability of the system was evaluated. Only L-Arg–containing formulations produced substantial NO under oxidative conditions, whereas Fc alone showed no detectable NO production (Fig. 2G). Importantly, the coexistence of ROS (derived from β-lapa redox cycling and Fc-mediated reactions) and NO enabled the formation of reactive nitrogen species (RNS). This was confirmed using L-tyrosine as a probe, where a pronounced fluorescence signal at 420 nm was observed exclusively in the FSAL group, indicating efficient peroxynitrite (ONOO⁻) generation via the reaction between NO and ROS (Fig. 2H). Finally, the tumor-responsive drug release behavior of HFSAL was investigated. Owing to the disulfide bonds in the FSA backbone, the nanoplatform exhibited glutathione (GSH)-responsive characteristics. In the absence of GSH, negligible release of Fc or β-lapa was detected, whereas increasing GSH concentrations triggered rapid nanoparticle disassembly and a pronounced time- and concentration-dependent release profile (Fig. 2I, J). Taken together, these results demonstrate that HFSAL is a well-defined, stable, and tumor-responsive nano-prodrug capable of orchestrating a cascade ROS–RNS amplification process, thereby providing a robust physicochemical and mechanistic foundation for subsequent antitumor applications.

### HFSAL induces ROS-RNS cascade amplification and oxidative damage-mediated tumor cell death

**Fig. 3:**
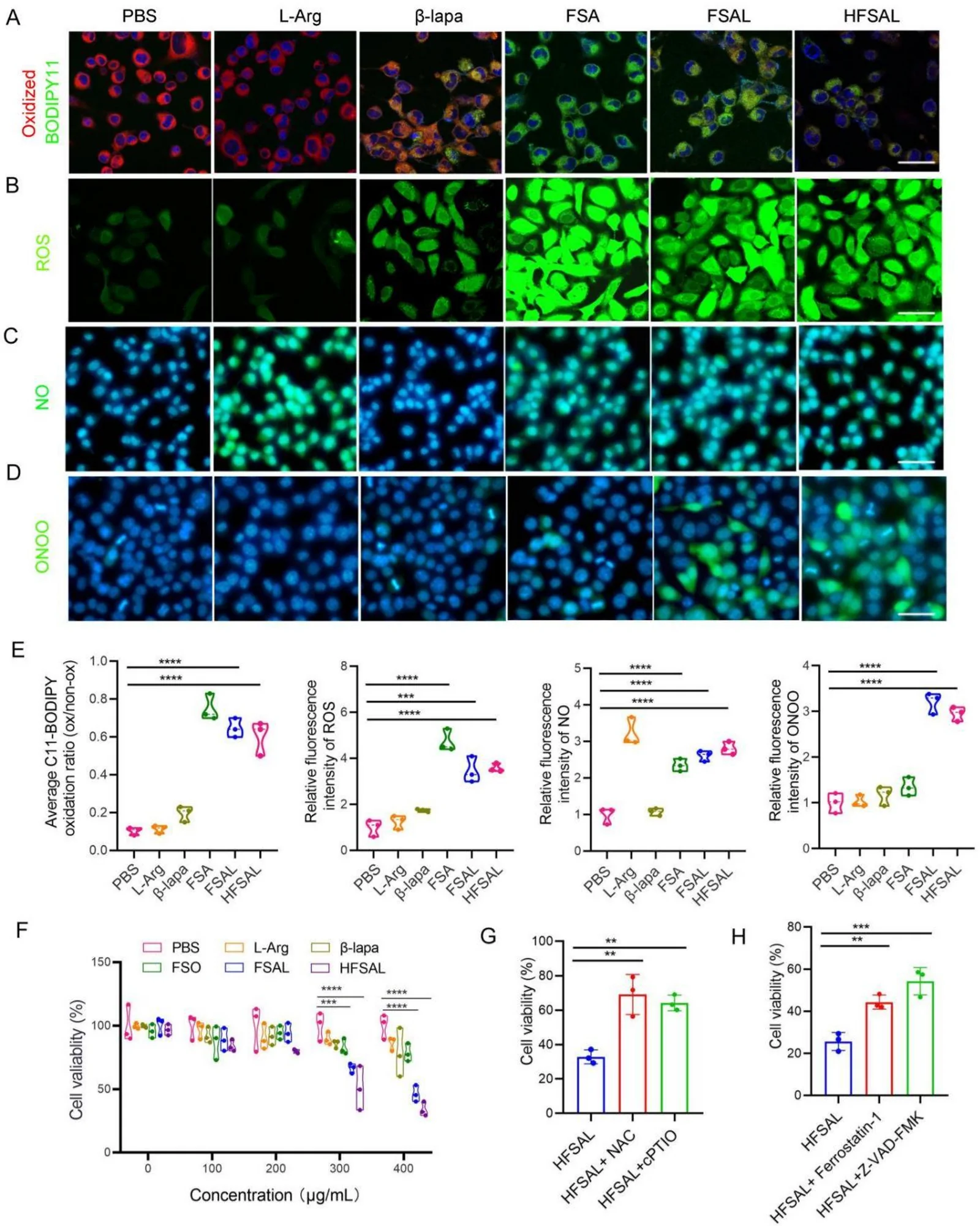
HFSAL amplifies intracellular ROS–RNS cascades and induces oxidative damage–mediated tumor cell death in vitro. (**A**) Fluorescence images of lipid peroxidation in B16F10 cells detected using C11-BODIPY after treatment with PBS, L-Arg, β-lapa, FSA, FSAL, or HFSAL (300 µg/mL). Oxidized C11-BODIPY (green) indicates lipid peroxidation. Scale bars, 50 μm. (**B–D**) Fluorescence imaging of intracellular reactive species in B16F10 cells after treatment with the indicated formulations (300 µg/mL). (**B**) ROS levels detected by DCFH-DA (green). (**C**) NO production detected using a NO-sensitive probe (blue). (**D**) ONOO⁻ levels detected using a peroxynitrite probe (green). Scale bars, 50 μm. (**E**) Quantitative analysis of lipid peroxidation (C11-BODIPY oxidation ratio) and fluorescence intensities of intracellular ROS, NO, and ONOO⁻ in B16F10 cells. Data are presented as mean ± s.d. (*n* = 3). (**F**) Cell viability of B16F10 cells treated with different formulations at indicated concentrations for 24 h. (**G**) Cell viability of B16F10 cells treated with HFSAL (300 µg/mL) in the absence or presence of N-acetylcysteine (NAC, 5 mM, ROS scavenger) or cPTIO (100 µM, NO scavenger) for 24 h. (**H**) B16F10 cells were pretreated with the ferroptosis inhibitor Ferrostatin-1 (Fer-1, 1 µM) or the pan-caspase apoptosis inhibitor Z-VAD-FMK (20 µM) for 2 h prior to HFSAL treatment (300 µg mL⁻¹). Cell viability was determined after 24 h using the MTT assay. Data are presented as mean ± s.d. (*n* = 3). Statistical significance was analyzed by one-way ANOVA (*P < 0.05, **P < 0.01, ***P < 0.001, ****P < 0.0001)

Building on the demonstrated redox activity and cascade reactivity of HFSAL at the physicochemical level, we next investigated whether this nanoplatform could effectively trigger intracellular oxidative stress and induce tumor cell death. We first assessed lipid peroxidation, a hallmark of ferroptosis, using C11-BODIPY staining. Compared with PBS and individual component treatments, HFSAL-treated B16F10 cells exhibited a markedly increased oxidized C11-BODIPY signal, indicating extensive lipid peroxidation (Fig. 3A). Notably, FSAL also induced lipid oxidation, whereas HFSAL further enhanced this effect, suggesting that HA coating and the optimized intracellular activation of the nanoplatform further strengthened oxidative damage. To elucidate the upstream mechanism, intracellular ROS levels were then examined. Fluorescence imaging revealed that HFSAL treatment led to a substantial elevation of intracellular ROS compared with all other groups (Fig. 3B), consistent with β-lapa-mediated redox cycling and Fc-driven Fenton reactions. In parallel, NO production was significantly increased in L-Arg-containing formulations, with the highest signal observed in the HFSAL group (Fig. 3C). Importantly, ONOO⁻ levels were dramatically elevated following HFSAL treatment (Fig. 3D), indicating efficient coupling between ROS and NO to generate highly reactive nitrogen species. Quantitative analysis further confirmed that HFSAL induced the highest levels of lipid peroxidation, ROS, NO, and ONOO⁻among all treatment groups (Fig. 3E), supporting the formation of a robust intracellular ROS–RNS cascade. The specificity and responsiveness of the fluorescence probes used for ROS, NO, and ONOO⁻detection were further validated using the corresponding positive controls (Rosup, NOup, and SIN-1, respectively). As shown in Fig. S9, HFSAL induced robust ROS, NO, and ONOO⁻signals comparable to those observed in the positive control groups, further supporting the activation of intracellular ROS-RNS cascade amplification. Importantly, the pronounced oxidative and nitrosative stress induced by HFSAL suggests a multifactorial cell death mechanism. Excessive ROS-RNS accumulation may simultaneously promote lipid peroxidation-driven ferroptosis and broader oxidative stress-associated apoptotic signaling, providing a mechanistic basis for synergistic tumor cell killing. We next evaluated whether this amplified oxidative stress translated into tumor cell death. HFSAL significantly reduced B16F10 cell viability in a dose-dependent manner and showed superior cytotoxicity compared with all other formulations (Fig. 3F). To confirm the contribution of ROS and NO, rescue experiments were performed using NAC and cPTIO. Both agents partially restored cell viability (Fig. 3G), indicating that ROS and NO are essential mediators of HFSAL-induced cytotoxicity. To further delineate the involvement of distinct cell death pathways, Ferrostatin-1 and Z-VAD-FMK were used to inhibit ferroptosis and apoptosis, respectively. As shown in Fig. 3H, both inhibitors significantly attenuated HFSAL-induced cytotoxicity, yet neither completely abolished the effect, suggesting cooperative involvement of ferroptosis and apoptosis in HFSAL-mediated tumor cell death. Collectively, these results demonstrate that HFSAL amplifies intracellular oxidative stress through coordinated ROS-RNS cascade signaling, leading to synergistic induction of ferroptosis- and apoptosis-associated tumor cell death.

### HFSAL exhibits enhanced tumor accumulation and induces transcriptional reprogramming associated with oxidative stress-mediated tumor suppression in vivo

Following the in vitro demonstration that HFSAL induces ROS-RNS cascade amplification and tumor cell death, the extended blood circulation was firstly evaluated in healthy mice after intravenous injection with indocyanine green (ICG)-labeled nano-prodrugs (ICG-FSAL and ICG-HFSAL) and monitoring of fluorescent emission of ICG-containing formulations in plasma samples at predetermined time points. Consequently, the pharmacokinetic parameters of ICG-FSAL and ICG-HFSAL were characterized and the blood circulation half-lives (*t*_1/2_ values) were about 2.64 h and 5.75 h, respectively (Fig. S10). In comparison, HFSAL showed significantly prolonged blood circulation time, which could be attributed to the surface coating of HA that shielded the positive charge of FSAL to improve the serum stability. In addition, we quantified Fe accumulation in tumor tissues after systemic administration of FSAL and HFSAL using ICP-OES. As shown in Fig. S11, treatment with HFSAL resulted in a significantly higher Fe content in tumors compared with FSAL treatment. This result indicates that the HA coating not only enhances the colloidal stability of the nano-prodrug during blood circulation but also facilitates active tumor targeting via CD44-mediated recognition, leading to more efficient delivery of the Fc moiety to the tumor site.

**Fig. 4:**
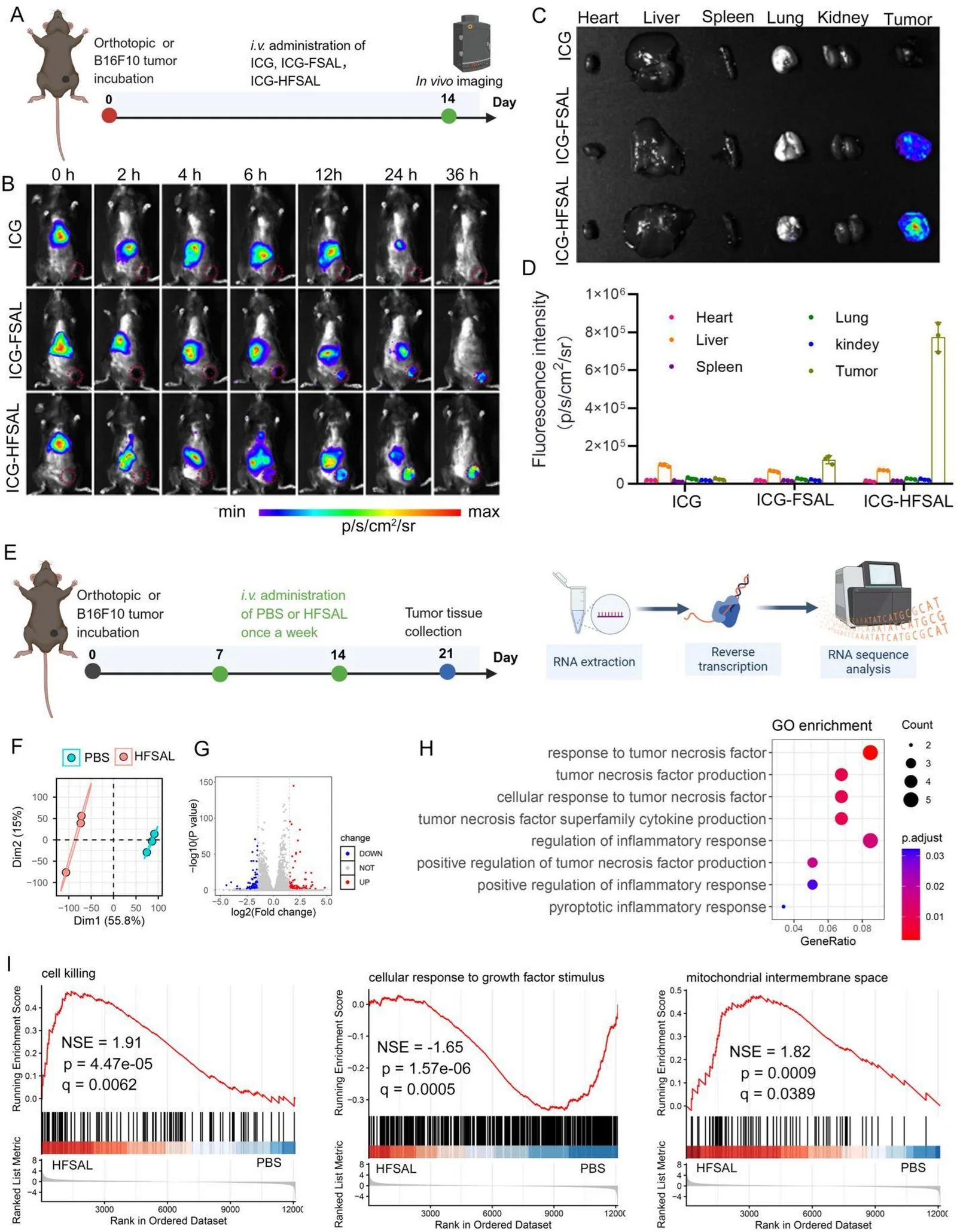
Tumor-targeted accumulation of HFSAL and transcriptional reprogramming in vivo. (**A**) Schematic illustration of in vivo imaging experiments in B16F10 tumor-bearing mice following intravenous administration of free ICG, ICG-FSAL, or ICG-HFSAL. (**B**) Representative in vivo fluorescence images of mice at different time points (0, 2, 4, 6, 12, 24, and 36 h) after intravenous injection of the indicated formulations. (**C**) Ex vivo fluorescence imaging of major organs (heart, liver, spleen, lung, kidney) and tumors collected at 36 h post-injection. (**D**) Quantitative analysis of fluorescence intensity in major organs and tumors at 36 h. Data are presented as mean ± s.d. (*n* = 3). (**E**) Schematic illustration of in vivo treatment and RNA sequencing workflow in B16F10 tumor-bearing mice. Mice were intravenously injected with PBS or HFSAL, followed by tumor collection for transcriptomic analysis. (**F**) Principal component analysis (PCA) of gene expression profiles showing distinct clustering between PBS- and HFSAL-treated tumors. (**G**) Volcano plot of differentially expressed genes (DEGs) between PBS and HFSAL groups (|log2FC| > 1.5, *P* < 0.05). (**H**) Gene Ontology (GO) enrichment analysis of upregulated DEGs in HFSAL-treated tumors. (**I**) Gene set enrichment analysis (GSEA) showing enrichment of pathways related to cell killing, cellular response to growth factor stimulus, and mitochondrial inner membrane space in HFSAL-treated tumors

Then, we next evaluated the in vivo tumor-targeting capability and therapeutic-associated transcriptional responses. To visualize biodistribution, free ICG and ICG-labeled nano-prodrugs (ICG-FSAL and ICG-HFSAL) were intravenously administered into B16F10 tumor-bearing mice (Fig. 4A). In vivo fluorescence imaging revealed that ICG-HFSAL exhibited significantly enhanced and sustained accumulation at tumor sites compared with free ICG and ICG-FSAL (Fig. 4B). While free ICG showed rapid clearance and limited tumor retention, HFSAL displayed prolonged circulation and gradual enrichment within tumors over time. Consistent with in vivo imaging results, ex vivo fluorescence analysis of major organs and tumors at 36 h post-injection demonstrated markedly higher signal intensity in tumors from the HFSAL group (Fig. 4C). Quantitative analysis further confirmed that HFSAL achieved superior tumor accumulation with relatively low off-target distribution (Fig. 4D), indicating effective tumor-targeted delivery.

To further explore the molecular mechanisms underlying HFSAL-mediated antitumor effects in vivo, we performed transcriptomic analysis of tumor tissues following treatment (Fig. 4E). Principal component analysis (PCA) revealed clear separation between PBS- and HFSAL-treated groups, indicating substantial transcriptional reprogramming induced by HFSAL (Fig. 4F). Differential expression analysis identified a large number of genes significantly altered upon HFSAL treatment (Fig. 4G). Gene Ontology (GO) enrichment analysis of upregulated genes revealed significant activation of biological processes associated with inflammatory response, cytokine production, and tumor necrosis factor signaling (Fig. 4H), suggesting that HFSAL may modulate the tumor microenvironment in addition to inducing oxidative stress. Furthermore, gene set enrichment analysis (GSEA) demonstrated significant enrichment of pathways related to cell killing, stress responses, and mitochondrial function in HFSAL-treated tumors (Fig. 4I). Notably, pathways associated with apoptosis and ferroptosis were also enriched, consistent with the ROS–RNS cascade–induced lipid peroxidation observed in vitro. Taken together, these results indicate that HFSAL achieves efficient tumor accumulation in vivo and induces broad transcriptional reprogramming associated with oxidative stress, inflammation, and cell death pathways, thereby contributing to its potent antitumor activity.

### Evaluation of the anti-tumor efficiency in the B16F10 primary tumor model

**Fig. 5:**
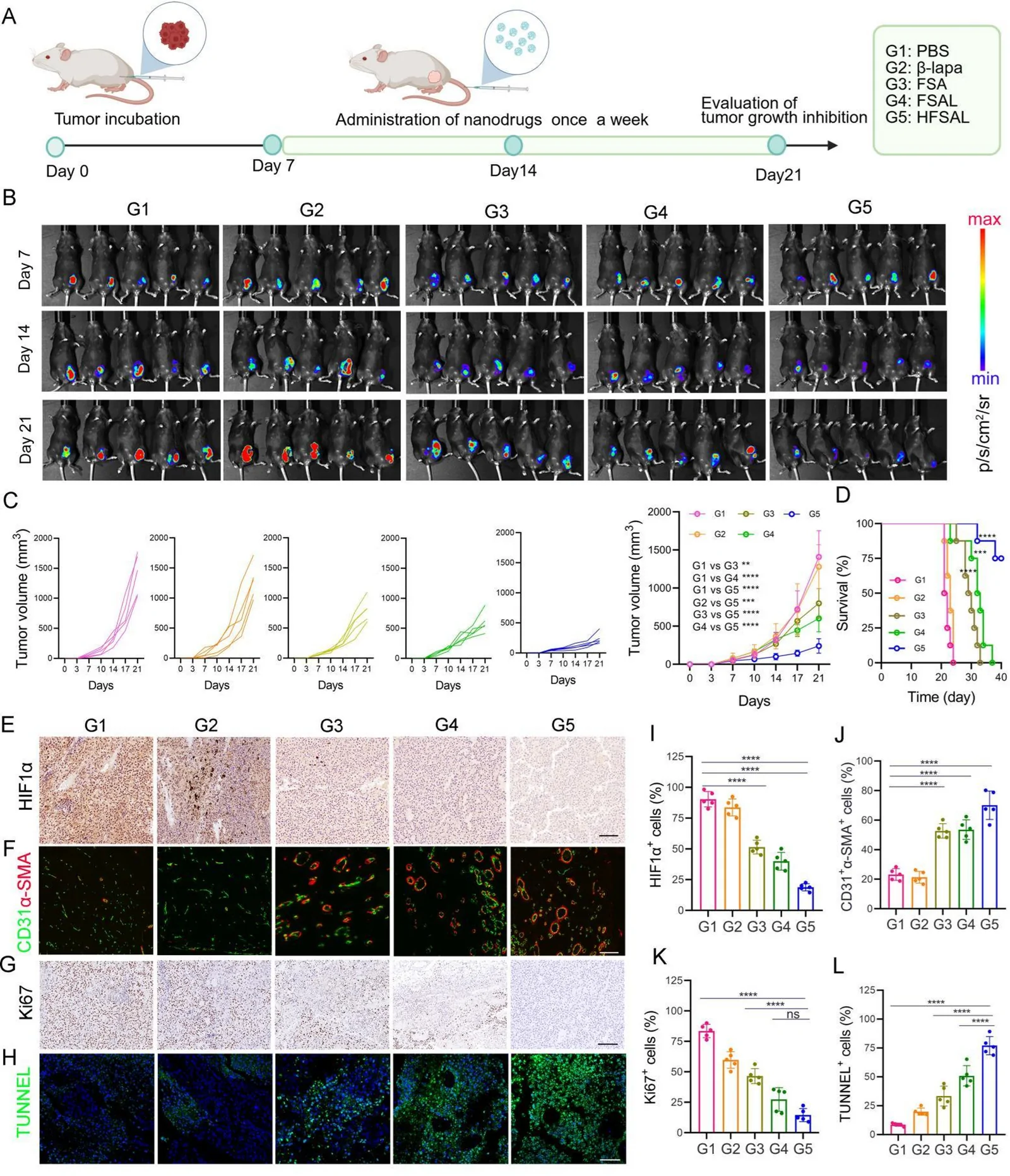
HFSAL exhibits potent antitumor efficacy in vivo through modulation of tumor hypoxia, vascular normalization, and tumor cell death. (**A**) Schematic illustration of the therapeutic protocol in B16F10 tumor-bearing mice. Mice were intravenously treated with PBS (G1), L-Arg (G2), FSA (G3), FSAL (G4), or HFSAL (G5) once per week, and tumor growth was monitored over time. (**B**) Representative in vivo bioluminescence images of tumor-bearing mice in different treatment groups at the indicated time points. Quantification of fluorescence intensity is shown on the right. Data are presented as mean ± s.d. (*n* = 5). Statistical data were analyzed by two-way ANOVA with Bonferroni post-hoc test. (**C**) Tumor growth curves of individual mice and average tumor volumes in different treatment groups over time. Data are presented as mean ± s.d. (*n* = 5). Statistical data were analyzed by two-way ANOVA with Bonferroni post-hoc test. (**D**) Kaplan-Meier survival curves of mice in different treatment groups (*n* = 8 ). Statistical significance was analyzed using the log-rank test. (**E**) Representative immunohistochemical staining of HIF-1α in tumor sections, indicating tumor hypoxia status. Scale bar, 50 μm. (**F**) Immunofluorescence staining of CD31 (endothelial marker) and α-SMA (pericyte marker) in tumor tissues to evaluate vascular normalization. Scale bar, 50 μm. (**G**) Representative immunohistochemical staining of Ki67 in tumor sections to assess cell proliferation. Scale bar, 50 μm. (**H**) TUNEL staining of tumor sections to evaluate apoptosis. Scale bar, 50 μm. (**I-L**) Quantitative analysis of HIF-1α-positive cells, CD31⁺α-SMA⁺vessel coverage, Ki67-positive cells, and TUNEL-positive cells in tumor tissues. Data are presented as mean ± s.d. (*n* = 3). Statistical significance was analyzed by one-way ANOVA (*P < 0.05, **P < 0.01, ***P < 0.001, ****P < 0.0001)

To evaluate the in vivo therapeutic efficacy of the nanoplatform, B16F10 tumor-bearing mice were treated with PBS (G1), β-lapa (G2), Fc-SS-Arg (FSA, G3), FSAL (G4), or HFSAL (G5) according to the schedule shown in Fig. 5A. Bioluminescence imaging revealed rapid tumor progression in the PBS group, whereas β-lapa alone showed minimal therapeutic benefit (Fig. 5B, Fig. S12). In contrast, FSA treatment moderately suppressed tumor growth, while FSAL exhibited enhanced tumor inhibition. Notably, HFSAL achieved the most pronounced suppression of tumor burden, as evidenced by markedly reduced bioluminescence signals compared with all other groups. These findings were further supported by tumor growth curves (Fig. 5C) and survival analysis, in which HFSAL-treated mice displayed the slowest tumor progression and the longest survival (Fig. 5D). To further assess the translational potential of HFSAL, we compared its therapeutic efficacy with the clinically used immune checkpoint inhibitor anti-PD-1 and evaluated their combination in B16F10 tumor-bearing mice. As shown in Fig. S13, anti-PD-1 monotherapy exhibited moderate tumor growth inhibition, whereas HFSAL treatment achieved superior antitumor efficacy. Notably, the combination of HFSAL and anti-PD-1 further suppressed tumor progression and prolonged survival compared with either monotherapy alone, suggesting a potential synergistic interaction between redox-mediated tumor killing and immune checkpoint blockade. These findings highlight the translational relevance of HFSAL and support its potential application in combination with current standard-of-care immunotherapy for melanoma. To investigate the underlying mechanisms, tumor sections were subjected to histological and immunofluorescence analyses. HIF-1α staining revealed that HFSAL treatment significantly alleviated tumor hypoxia compared with all other groups (Fig. 5E, I). Consistently, vascular normalization was observed in the HFSAL group, as indicated by increased α-SMA⁺/CD31⁺ colocalization (Fig. 5F, J). In addition, HFSAL markedly suppressed tumor cell proliferation, as demonstrated by reduced Ki67⁺ staining (Fig. 5G, K), and induced extensive apoptosis, evidenced by increased TUNEL⁺ signals (Fig. 5H, L). Consistent with these histological findings, western blot analysis of tumor tissues further confirmed reduced HIF-1α and Ki67 expression, accompanied by increased cleaved caspase-3 levels in the HFSAL-treated group (Fig. S14A), supporting the alleviation of hypoxia, inhibition of tumor proliferation, and induction of apoptosis. These effects were more pronounced than those observed in the FSA and FSAL groups. Finally, the biosafety of the treatments was systematically evaluated. H&E staining of major organs showed no observable histopathological abnormalities across all groups (Fig. S15). Serum biochemical parameters, including LDH, BUN, ALT, AST, and ALP, remained within normal ranges (Fig. S16), and no significant body weight loss was observed during treatment (Fig. S17). Collectively, these results demonstrate that HFSAL exerts potent anti-tumor activity in vivo while maintaining a favorable safety profile.

### Evaluation of the anti-tumor efficiency in B16F10 metastatic tumor model

**Fig. 6:**
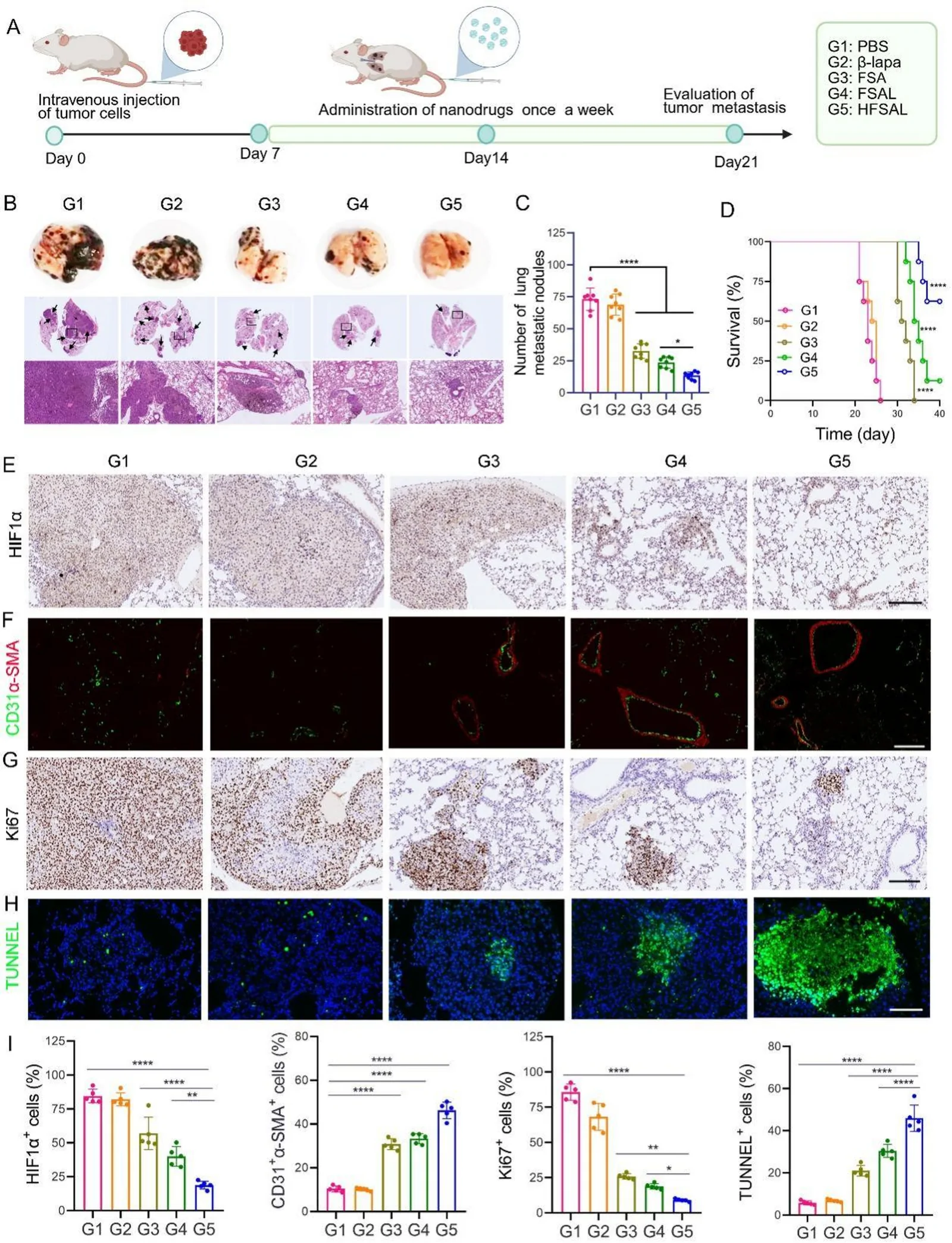
HFSAL suppresses lung metastasis and modulates the tumor microenvironment in a B16F10 metastatic model. (**A**) Schematic illustration of the experimental design for evaluating anti-metastatic efficacy in B16F10 lung metastasis model. Tumor cells were intravenously injected, followed by systemic administration of different formulations.(**B**) Representative photographs and H&E-stained sections of lungs collected from different treatment groups, showing metastatic tumor nodules. (**C**) Quantitative analysis of the number of metastatic lung nodules in each group. Data are presented as mean ± s.d. (*n* = 8). Statistical significance was analyzed by one-way ANOVA. (**D**) Kaplan–Meier survival curves of mice bearing metastatic tumors (*n* = 8). Statistical significance was analyzed using the log-rank test. (**E**) Representative immunohistochemical staining of HIF-1α in metastatic lung tumors, indicating hypoxia levels. Scale bar, 50 μm. (**F**) Immunofluorescence staining of CD31 (endothelial marker) and α-SMA (pericyte marker) in metastatic lung tumors to evaluate vascular normalization. Scale bar, 50 μm. (**G**) Representative immunohistochemical staining of Ki67 in metastatic lung tumors to assess cell proliferation. Scale bar, 50 μm. (**H**) TUNEL staining of metastatic lung tumors to evaluate apoptotic cell death. Scale bar, 50 μm. (**I**) Quantitative analysis of HIF-1α-positive cells, CD31⁺α-SMA⁺ vessel coverage, Ki67-positive cells, and TUNEL-positive cells in metastatic lung tumors. Data are presented as mean ± s.d. (*n* = 3). Statistical significance was analyzed by one-way ANOVA (*P < 0.05, **P < 0.01, ***P < 0.001, ****P < 0.0001)

Building on the promising tumor growth inhibition observed in the primary tumor model, the anti-tumor activity of HFSAL was further evaluated in the metastatic B16F10 tumor model. The B16F10 metastatic tumor-bearing mice were established *via* tail vein injection of B16F10 cells and subsequently randomized into five treatment groups. Mice were treated twice a week for two weeks with the indicated formulations (Fig. 6A). Obvious metastatic tumor nodules on the surface of the lungs were observed after the treatment by control and β-lapa (Fig. 6B, C). In contrast, HFSAL treatment significantly reduced the number of lung metastatic lesions, as evidenced by representative photographs and H&E-stained sections of metastatic lung tissues (Fig. 6B, C). FSAL also reduced tumor metastasis compared to the control group, although its anti-metastatic effect was less pronounced than that of the HFSAL nanosystem. Notably, HFSAL treatment conferred the longest survival time, underscoring its potent therapeutic efficacy (Fig. 6D). To investigate the underlying mechanisms, we first assessed the expression of HIF-1α and α-SMA⁺/CD31⁺in lung metastatic tumors. HFSAL and FSAL treatments markedly reduced HIF-1α expression and promoted vascular normalization with enhanced α-SMA⁺/CD31⁺ colocalization compared to the control, while β-lapa treatments did not significantly alter HIF-1α levels and α-SMA⁺/CD31⁺expression (Fig. 6E, F). This suggests that the NO released from L-Arg within the nano-prodrugs alleviated tumor hypoxia and promoted vascular normalization. The Ki67 and TUNNEL assays were then performed to evaluate the proliferation and apoptosis levels in the metastatic tumor microenvironment. As shown in Fig. 6G, H, the proliferation rate (discerned by Ki67 staining) was lowest by HFSAL treatment, whereas the percentage of apoptosis cells (TUNNEL staining) was highest in tumor sections. Consistent trends were observed in metastatic tumors by western blot analysis (Fig. S14B), supporting the alleviation of hypoxia, inhibition of tumor proliferation, and induction of apoptosis. Together, these results suggest the nano-drug system we have developed is effective for treating metastatic tumor model.

## Conclusion

In summary, we developed a GSH-responsive, self-assembled nano-prodrug (HFSAL) that integrates ferrocene, β-lapachone, and L-arginine into a single platform to achieve coordinated ROS-RNS cascade amplification for cancer therapy. By rationally combining NQO1-activated redox cycling, Fenton chemistry, and NO generation, HFSAL effectively overcomes key limitations of conventional ferroptosis-based strategies, including insufficient oxidative stress induction, limited Fe^2+^ availability, and suboptimal tumor selectivity. Upon tumor accumulation and intracellular activation, β-lapa amplifies endogenous ROS production through NQO1-dependent redox cycling, Fc catalyzes hydroxyl radical generation via Fenton reactions, and L-Arg-derived NO promotes the formation of highly cytotoxic reactive nitrogen species, particularly ONOO-. This coordinated redox network results in amplified oxidative damage and synergistically induces ferroptotic and apoptotic cell death. The coordinated activation of these two regulated cell death pathways is likely driven by excessive ROS-RNS accumulation, which simultaneously promotes lipid peroxidation and caspase activation. In addition, RNS generation contributes to tumor microenvironment remodeling by alleviating hypoxia and promoting vascular normalization, thereby facilitating improved intratumoral drug delivery and therapeutic efficacy. This microenvironment remodeling may also enhance responsiveness to immune checkpoint blockade, which is consistent with the improved therapeutic efficacy observed when HFSAL was combined with anti-PD-1 therapy. Comprehensive in vitro and in vivo evaluations demonstrated that HFSAL effectively suppresses tumor growth in both primary and metastatic melanoma models while maintaining a favorable safety profile. Transcriptomic analyses further supported the activation of redox- and cell death-associated pathways, highlighting the mechanistic synergy of this nanoplatform. Although the present study focuses on melanoma, the proposed strategy may also be applicable to other NQO1-overexpressing malignancies. Future studies should further evaluate large-scale manufacturing, long-term biosafety, and therapeutic efficacy in additional clinically relevant models to facilitate clinical translation. Overall, this work establishes a redox-amplifying nanoplatform that integrates ROS generation, Fe^2+^ supply, and RNS-mediated cytotoxicity within a single programmable system. By leveraging the interplay between ferroptosis and apoptosis, this strategy provides a versatile and effective framework for the treatment of aggressive and therapy-resistant cancers.

## Supplementary Information

Supplementary Material 1

## Data Availability

No datasets were generated or analysed during the current study.
