# Supplementary material for: Cascade-activatable nano-prodrug amplifies ROS-RNS signaling to target NQO1-associated redox vulnerability for melanoma therapy
Source: J Nanobiotechnology. 2026 Aug 3;24:1009. doi: 10.1186/s12951-026-04825-0 (PMC13721699; doi:10.1186/s12951-026-04825-0)
Supplement: Supplementary file 1 [file 12951_2026_4825_MOESM1_ESM.docx]

**Supporting information**

**Cascade-Activatable Nano-Prodrug Amplifies ROS-RNS Signaling to Target NQO1-Associated Redox Vulnerability for Melanoma Therapy**

Honglin Tang^a,1^, Chongzhi Wu^b,1^, Wei Wang^a^, Xi Cheng^a^, Yong Dong^a^, Zhiyuan Gao ^b^, Pengfei Chen^b^, Xin Xie^c^, Yong Huang ^c^, Lihe Sun^c*^, Bowen Li^b*^, Da Li^a*^

a Department of Medical Oncology Sir Run Run Shaw Hospital School of Medicine, Zhejiang University, Hangzhou 310058, China.

b School of Pharmacy, Anhui Medical University, Hefei 230032, P. R. China

c School of Pharmacy, Guangdong Medical University, Dongguan 523808, China.

*Corresponding authors.

E-mail addresses: lidaonconew@zju.edu.cn, li860316@163.com, sun.lihe@gdmu.edu.cn

^1^ These authors contributed equally to this work.

**Scheme S1.** Synthetic route for FSA.

**Fig. S1.** ^1^H NMR spectrum of FSO in CDCl_3_.

**Fig. S2.** ^1^H NMR spectrum of Fc-SS-Arg(Boc) in CDCl_3_.

**Fig. S3.** HR-MS result of Fc-SS-Arg(Boc). ESI-MS (*m/z*): 823.26694 [M+H]^+^.

**Fig. S4.** ^1^H NMR spectrum of FSA in CDCl_3_.

**Fig. S5.** HR-MS result of FSA. ESI-MS (*m/z*): 523.11212 [M+H]^+^.

**Fig. S6** The ·OH generation of PBS and FSA under H_2_O_2_ stimulation at different pH environment.

**Fig. S7.** The particle size and size distribution tracking of FSAL within 15 days under physiological conditions (PBS containing 10% FBS).

**Fig. S8.** The particle size and size distribution tracking of HFSAL within 15 days under physiological conditions (PBS containing 10% FBS).

**Fig. S9.** Validation of fluorescence probes for ROS, NO, and ONOO⁻ detection using positive controls. (A) Representative confocal fluorescence images of intracellular ROS, NO, and ONOO⁻ levels in B16F10 cells treated with PBS, HFSAL, or the corresponding positive controls. Rosup was used as a positive control for ROS generation, NOup was used as a positive control for NO generation, and SIN-1 was used as a positive control for ONOO⁻ generation. Scale bar = 50 μm. (B) Quantitative analysis of intracellular ROS, NO, and ONOO⁻ fluorescence intensities in different treatment groups. Data are presented as mean ± SD (n = 3 biologically independent experiments). Statistical significance was analyzed using one-way ANOVA followed by Tukey’s multiple-comparison test. ***P < 0.001, ****P < 0.0001.

**Fig. S10.** Blood circulation of ICG-FSAL or ICG-HFSAL after tail-vein injection in healthy mice.

**Fig. S11.** Fe accumulation in tumor tissues at predetermined time points post intravenous injection of FSAL or HFSAL nano-prodrugs (equivalent Fe dosage at 100 μg/mouse).

**Fig. S12.** Mice were intravenously treated with PBS (G1), L-Arg (G2), FSA (G3), FSAL (G4), or HFSAL (G5) once per week, and tumor growth was monitored over time. Quantification of fluorescence intensity is shown on the right. Data are presented as mean ± s.d. (n = 5). Statistical data were analyzed by two-way ANOVA with Bonferroni post-hoc test. (*P < 0.05, **P < 0.01, ***P < 0.001, ****P < 0.0001).

**Fig. S13**. HFSAL in combination with anti-PD-1 enhances in vivo antitumor efficacy in B16F10 tumor-bearing mice. (A) Representative images of excised tumors from mice treated with PBS, anti-PD-1, HFSAL, or the combination of HFSAL and anti-PD-1. Tumors were collected on day 21 post-treatment. (B) Tumor growth curves of the different treatment groups over 21 days. HFSAL monotherapy significantly inhibited tumor growth compared with PBS and anti-PD-1 alone, while the combination treatment exhibited the strongest tumor suppression. Data are presented as mean ± SD (n = 5). Statistical significance was determined by two-way ANOVA with Bonferroni post-hoc test (****P < 0.0001).

**Fig S14**. Western blot validation of hypoxia, proliferation, and apoptosis markers in tumor tissues from B16F10 subcutaneous and lung metastatic models.(A) Representative western blots of HIF-1α, Ki67, and cleaved caspase-3 in tumors from the B16F10 subcutaneous model treated with PBS, L-Arg, FSA, FSAL, or HFSAL. GAPDH was used as the loading control. HFSAL treatment reduced HIF-1α and Ki67 expression while increasing cleaved caspase-3 levels, consistent with immunofluorescence and TUNEL staining results.(B) Representative western blots of HIF-1α, Ki67, and cleaved caspase-3 in lung metastatic tumors from the B16F10 model under the same treatment groups. Results are consistent with the subcutaneous tumor data, supporting alleviation of hypoxia, suppression of proliferation, and induction of apoptosis by HFSAL. Experiments were repeated at least three times with consistent results. Representative blots are shown.

**Fig. S15** Representative H&E-stained sections of major organs collected at day 21 from mice treated with PBS (G1), L-Arg (G2), FSA (G3), FSAL (G4), or HFSAL (G5). Scale bar = 50 μm.

**Fig. S16** Serum biochemistry analysis, including lactate dehydrogenase (LDH), blood urea nitrogen (BUN), alanine aminotransferase (ALT), aspartate aminotransferase (AST), and alkaline phosphatase (ALP).

**Fig. S17** Body temperature profiles of B16F10 tumor-bearing mice during treatment with the indicated formulations.
